# Impact of the COVID-19 pandemic on perceived access and quality of care in German people with parkinsonism

**DOI:** 10.3389/fpubh.2023.1091737

**Published:** 2023-04-14

**Authors:** Marlena van Munster, Marcel R. Printz, Eric Crighton, Tiago A. Mestre, David J. Pedrosa, iCARE-PD Consortium

**Affiliations:** ^1^Department of Neurology, Philipps University Marburg, Baldingerstraße, Marburg, Germany; ^2^Department of International Health, Maastricht University, CAPHRI Care and Public Health Research Institute, Maastricht, Netherlands; ^3^Department of Geography, Environment and Geomatics, University of Ottawa, University Private, Ottawa, ON, Canada; ^4^Parkinson’s Disease and Movement Disorders Clinic, Department of Medicine, The Ottawa Hospital Research Institute, The University of Ottawa Brain and Mind Research Institute, Ottawa, ON, Canada; ^5^Centre of Mind, Brain and Behaviour, Philipps University Marburg, Hans Meerwein Straße, Marburg, Germany

**Keywords:** Parkinson’s disease, COVID-19 pandemic, health care, impact, Germany, access

## Abstract

Due to the heterogeneous clinical presentation, people with Parkinsonism (PwP) develop individual healthcare needs as their disease progresses. However, because of limited health resources during the COVID-19 pandemic, many patients were put at risk of inadequate care. All this occurred in the context of inequitable healthcare provision within societies, especially for such vulnerable populations. This study aimed to investigate factors influencing satisfaction and unmet need for healthcare among PwP during the COVID-19 pandemic in Germany. Analyses relied on an anonymous online survey with a 49-item questionnaire. We aimed at describing access to health services before and during the early stages of the pandemic. To this end, a generalized linear model was used to derive significant predictors and a stepwise regression to subsummarize the main factors of perceived inadequate care. In total, 551 questionnaires showed that satisfaction with Parkinsonism-related care decreased significantly during the pandemic (*p* < 0.001). In particular, factors such as lower educational level, lower perceived expertise of healthcare providers, less confidence in remote care, difficulties in obtaining healthcare, and restricted access to care before the pandemic but also lower densities of neurologists at residence and less ability to overcome barriers were indicative of higher odds to perceive unmet needs (*p* < 0.05). The results unveil obstacles contributing to reduced access to healthcare during the COVID-19 pandemic for PwP. These findings enable considerations for improved provision of healthcare services to PwP.

## 1. Introduction

The COVID-19 pandemic presented unprecedented challenges worldwide afflicting people adversely, economically, and culturally. In response to rising caseloads, public life was shut down, and access to health services, among others, was disrupted (1–3). Scientific evidence suggests that healthcare utilization declined by approxiamtely one-third during the pandemic (2). In Germany, a decrease in the use of outpatient and inpatient services was reported during the first wave, with dental and specialist examinations being canceled most frequently, followed by physiotherapy, occupational therapy, or speech therapy (4). Yet, this disruption affected individuals in Germany to varying degrees and especially those with chronic diseases, such as persons with Parkinson’s disease (PD) or Parkinsonism (5–8). This is not too surprising, as persons with PD belong to the high-risk group for severe disease or for secondary complications of COVID-19, which made them reluctant to visit medical facilities (9).

Patients with Parkinsonism show a progressive condition characterized by motor but also non-motor symptoms. A plethora of different clinical signs may emerge during the disease’s course, requiring continuous therapy adjustments and need assessments by healthcare professionals. Parkinsonism negatively affects individual psychosocial functioning (10), often leaving those affected in need of social, financial, or physical support. People suffering from chronic diseases, including persons with Parkinsonism, often necessitate continuous medical services outside of emergency departments, such as frequent physiotherapy, and therefore appeared at high risk of undersupply during the pandemic (1, 3, 7). Recent studies have unveiled the impact of the COVID-19 pandemic on people suffering from PD (6, 11–14). For the German population, Zipprich et al. (11) interviewed people with PD about their experience of healthcare during the pandemic. Approximately one-third indicated that they experienced a decrease in their mobility because regular therapies (e.g., physiotherapy) were canceled. Fründt et al. (12) also showed that people with PD who received long-term care were more socially isolated during the pandemic than those who did not receive long-term care. Thus, it seems likely that people with PD were affected to varying degrees by the constraints during the pandemic, not least because other areas of public health research also suggest that health crises have a highly individualized impact on access to care for vulnerable groups (15–17).

Beyond the variable degree of disability due to Parkinsonism, other variables may influence how severely access to healthcare may be inferred. These variables are also described as determinants. Determinants of access to healthcare may pose interesting concepts to identify individuals who are particularly restricted in their access to healthcare by a public health crisis. What can be considered a relevant determinant, however, is by no means universal, and rather context-specific considerations are required (18). For PD, Zaman et al. (19) proposed a model summarizing structural and individual factors potentially influencing patients’ access to healthcare. Structural determinants encompass barriers that patients meet on a system level when accessing healthcare, such as a lack of care coordination, limited communication between healthcare providers, disparities in health services, or the unavailability of specialized services (19). Individual barriers, such as available financial resources, influence patients’ abilities to seek help or to engage with care providers and to reach out to important care services (19), which may likewise be of great importance. In particular, patients often hinge on a good support network.

To our knowledge, it has not yet been investigated how determinants of access to healthcare may relate to the perceived healthcare situation during the COVID-19 pandemic of persons with Parkinsonism in Germany. Therefore, we examined the impact of a multitude of factors on this population with special emphasis on their access to healthcare.

## 2. Methods

We conducted a cross-sectional, anonymous online survey of persons with Parkinsonism (PwP) in Germany (or their caregivers). There were no exclusion criteria regarding disease duration or severity. Participants at all stages of the disease were eligible to participate in the survey. The survey consisted of a questionnaire, which was distributed nationwide using the members’ email newsletter of the German Parkinson Association (Deutsche Parkinson Vereinigung e.V., dPV) between November 2020 and January 2021. The newsletter is a free offer of the dPV e.V. and is sent out at regular intervals *via* its own email distribution list. It contains information from the association as well as a part about the latest research projects. The invitation to the survey consisted of a short description with a link to an online survey, which patients could access using a personal computer, a tablet, or a smartphone. In Germany, SoSci Survey (20) served as a database for hosting the survey. Throughout the data input, the database was supervised and manually checked for plausibility. The study was approved by the local ethics committee (reference number: AZ 164/19) and carried out in accordance with the Declaration of Helsinki. All patients gave informed written consent before participating.

### 2.1. Questionnaire

This study was carried out as part of the multinational iCARE-PD-project.^1^ Within the scope of this project, a 49-item questionnaire was developed which aimed at characterizing the access of PwP to healthcare services before and during the pandemic. In addition to Germany, the iCARE-PD questionnaire was also shared with patient associations in Canada, Spain, Portugal, and the Czech Republic with the respective translations. In this study, we limited ourselves to data collected from German patients. For that purpose, the initial questions in English were translated to German and were structured in four sections: (A) patients’ health status in terms of Parkinsonism, operationalized by (21) and (22), but also concomitant diseases, (B) experiences with healthcare services within 12 months before the pandemic, (C) experiences with healthcare services during the COVID-19 pandemic with special emphasis on telemedicine services, and (D) demographic and socioeconomic characteristics of participants. There were single and multiple-choice questions along with open-ended questions, some of which depended upon the specific answers to previous ones. A full version of the questionnaire is included in the Supplementary Material.

### 2.2. Statistical analyses

**Figure 1:**
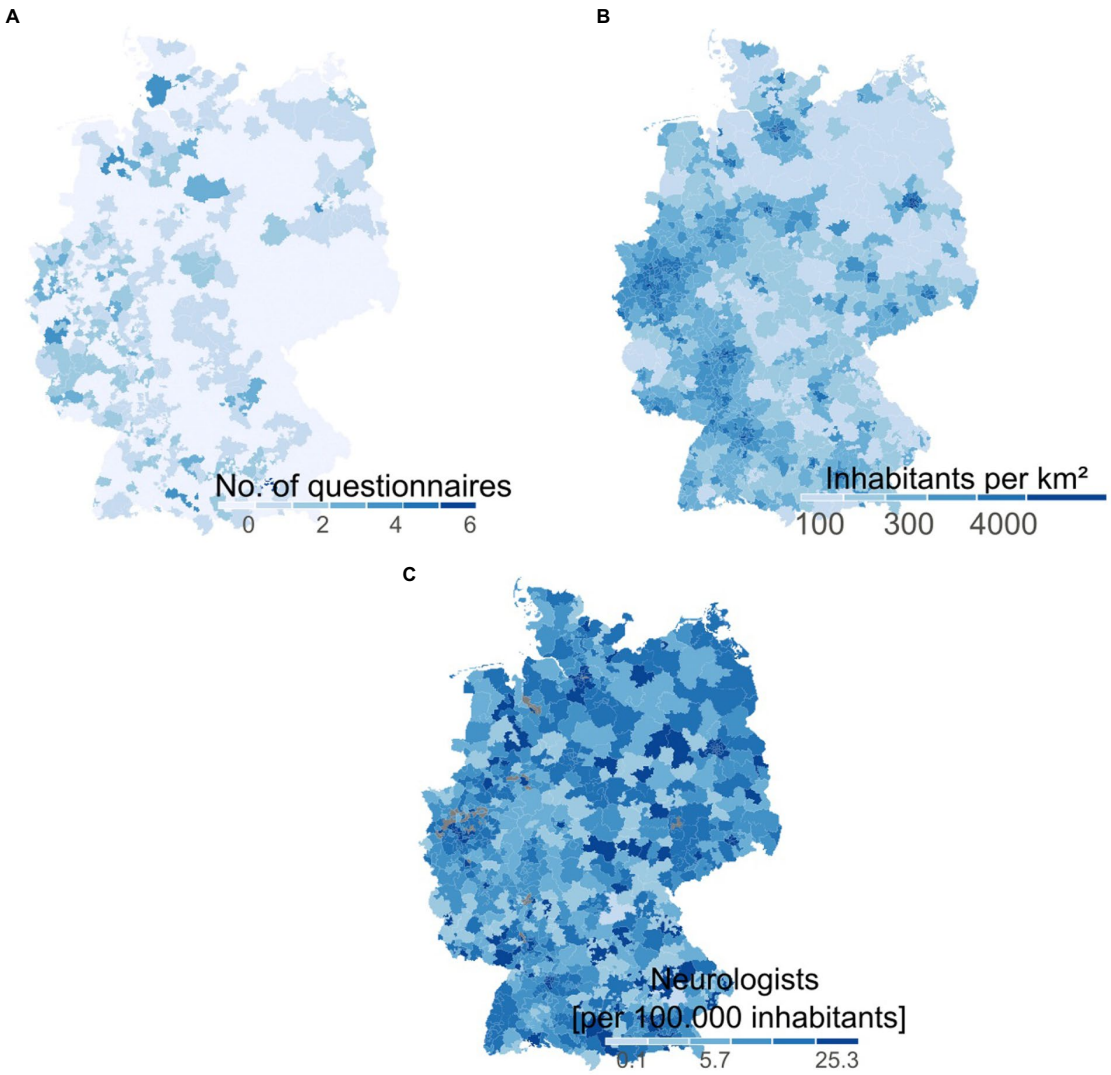
Demographic data for Germany and additional regional data for the obtained questionnaires. **(A)** Number of received questionnaires within our survey for the distinct three-digit postal codes. **(B)** Illustration of inhabitants per square kilometer for Germany. **(C)** Density of neurologists in all parts of Germany according to the German Statutory Health Insurance Association (Kassenärztliche Bundesvereinigung).

All analyses were conducted in R (23). Publicly available data on population densities^2^ and those for neurologists^3^ could be added to the analyses for regional data containment. For that purpose, we used the first three numbers of their German postal code, which were disclosed in the last section of the survey. Merging the available data with the maps for postal codes^4^ resulted in regional distributions (*cf.* Figure 1).

Population and neurologist densities were stratified into five equal quantiles for further analyses. Moreover, the provided information on concomitant diseases (in addition to Parkinsonism) was collated to a score—the Elixhäuser Comorbidity Score with its modification introduced by van Walraven et al. (24) with higher values indicating a more severe disease burden. Finally, all questions were assigned to barriers to accessing health services regarding Parkinsonism as described by (19) (*cf.* Supplementary Table S1). After the estimation of descriptive statistics, satisfaction with overall Parkinson’s-related care was compared before and during the pandemic using a non-parametric sign test (rstatix package^5^). The two questions that were used are as follows:

“In the 12 months prior to the COVID-19 pandemic, overall, how satisfied were you with the way healthcare services related to Parkinsonism were provided?” (B17)

vs.

“Since the beginning of the COVID-19 pandemic, overall, how satisfied are you with the way healthcare services related to Parkinsonism are provided?” (C6)

Furthermore, using a generalized linear model (GLM) with a binomial link function, we estimated odds ratios for worse satisfaction with Parkinsonism-related care. After establishing the full model with a total of 32 predictors, we conducted a stepwise logistic regression to reduce the complexity, leaving the most meaningful predictors for the question: “Since the beginning of the COVID-19 pandemic, how often did you feel you needed healthcare for Parkinsonism but did not receive it?” (C4). For that, the first missing data were imputed by taking advantage of a multivariate imputation scheme using the Mice package (25). We thereby assumed data missing at random and used the predictive mean matching method. Consecutively, stepwise reduction using a GLM with stepwise feature selection (glmStepAIC) in both directions from the caret-package (26) aimed at minimizing the Akaike information criterion (AIC). We first split all available data into 80% of training and 20% of test data and performed the stepwise regression after centering and rescaling values and by applying 10-fold cross-validation. The predictions of the two models were compared with the test data using accuracy, area under the curve (AUC), and LogLoss as metrics.

## 3. Results

In total, 551 questionnaires (response rate of about 3%) were filled out with 252 different postal codes from all 16 German regions (Bundesländer, *cf.* Figure 1). Of all participants, 388 (70.4%) returned a complete questionnaire (for demographics from parts A and D, *cf.* Table 1).

**Table 1:** Demographics and clinical characteristics of survey respondents.

	Overall (*n* = 551)
Age [mean (SD)]	66.76 (9.25)
Gender = Female (%)	148 (41.6)
Time since Parkinsonism diagnosis (%)	
*<*2 years	62 (13.1)
2–5 years	154 (32.6)
5–10 years	157 (33.2)
10–15 years	69 (14.6)
*>*15 years	31 (6.6)
Disease stage (%)	
Hoehn & Yahr I	189 (40.3)
Hoehn & Yahr II	156 (33.3)
Hoehn & Yahr III	77 (16.4)
Hoehn & Yahr IV	41 (8.7)
Hoehn & Yahr V	6 (1.3)
Education level according to ISCED (%)	
Primary education	20 (5.0)
Secondary education	234 (58.4)
Post-secondary education	69 (17.2)
Highest education level possible	78 (19.5)
PDQ-8 scores [mean (SD)]	41.30 (14.23)
Van-Walraven-Elixhauser Comorbidity Index [mean (SD)]	6.55 (1.95)

**Figure 2:**
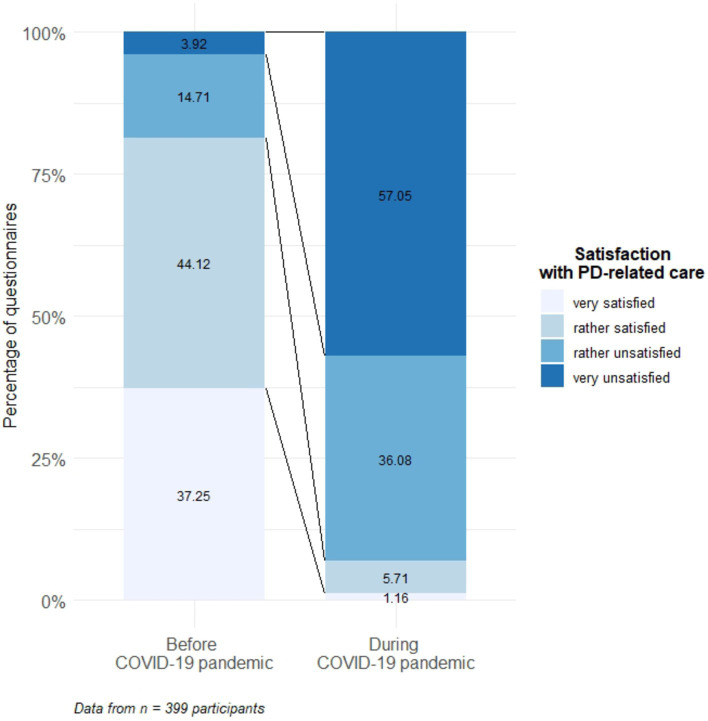
B17 vs. C6—Distribution of responses on satisfaction with Parkinsonism-related care before and during the COVID-19 pandemic.

Satisfaction for Parkinsonism-related care significantly decreased during the pandemic (pre-pandemic, Mdn = 3 vs. post-pandemic, Mdn = 1; *p* = 10–73). More than 90% of all participants stated to be somewhat unsatisfied or very unsatisfied with their Parkinsonism-related care during the pandemic (*cf.* Figure 2).

**Figure 3:**
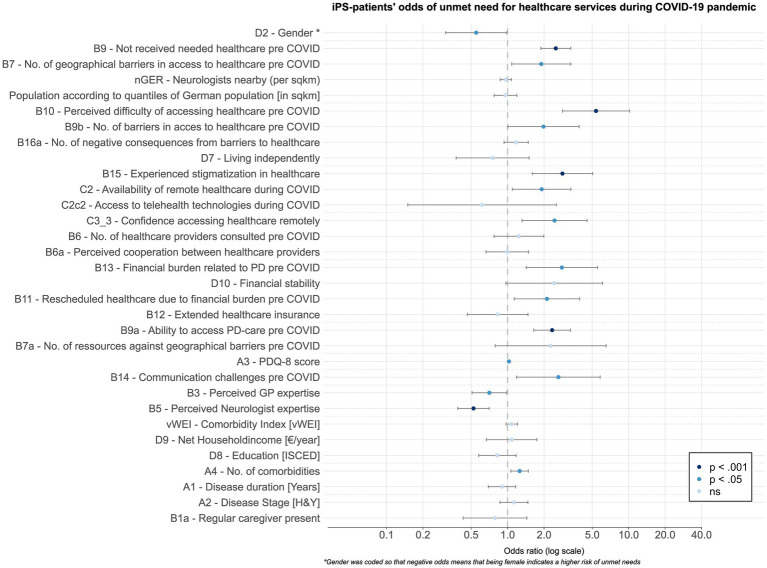
Odds ratios for all items in terms of perceived inadequate healthcare during the pandemic. Odds were determined *via* GLM and coded so that higher values indicate affirmation of the question that healthcare was needed but this need remained unmet during the COVID-19 pandemic. The dashed lines indicate the distinct domains according to Zaman et al., whereas significance is illustrated as the color of the dot, with two distinct levels of significance.

To ascertain factors associated with declines in satisfaction, logistic regressions on question C4 (“Since the beginning of the COVID-19 pandemic, how often did you feel you needed healthcare for Parkinsonism but did not receive it?”) were performed, unveiling factors which contribute to this perception of unmet needs during the pandemic (see Figure 3).

Thus, the odds to affirm this question were highly significant (*p* < 0.001) for those patients inferring lower levels of competence for their neurologist, with a lower ability to access Parkinsonism care before the pandemic, for patients with higher degrees of stigmatization in healthcare but also for those who did not receive healthcare services before the pandemic. A significant contribution—albeit lower with significance values of *p* < 0.05—was encountered for PwP with increasing levels of comorbidity, with perceived lower expertise of the general practitioner, and with a higher quality of life scores retrospectively, for people with higher financial burden due to Parkinsonism or who needed to reschedule healthcare appointments due to financial problems before the pandemic. Finally, the lack of availability of remote healthcare during the pandemic and geographical or in general more numerous barriers to accessing healthcare before the start of the pandemic was also indicative of higher odds to perceive unmet needs. For an illustration of significant predictors, see Figure 3, and for the entire list of results, *cf.* Table 2 in the Supplementary Material. In the assumption of an overfitted model, we performed a two-way stepwise regression for the question “C4” (see above) so that the initial 32 items could be reduced to seven significant predictors of unmet needs for healthcare services (*cf.* Table 2), namely:

- educational level
- perceived expertise of the general practitioner
- confidence in the ability to access required healthcare services remotely
- perceived ease of obtaining healthcare before the pandemic
- perceived availability of specialist care before the pandemic
- density of neurologists within the area of living
- availability of structural support to overcome the geographical barrier

**Table 2:** Significant factors contributing to unmet care needs during the COVID-19 pandemic according to the reduced GLM.

Predictor	Estimate	SE	*z*-value	*p*
(Intercept)	−2.65	0.29	−9.24	*<*0.0001
Educational level (D8)	−0.73	0.24	−3.01	0.003
Perceived GP’s expertise (B3)	0.34	0.17	2.07	0.038
Confidence in accessing necessary services remotely (C3)	0.64	0.22	2.90	0.004
Ease obtaining healthcare prior to the pandemic (B10)	−0.47	0.22	−2.15	0.031
Ability to access care prior to the pandemic (B9)	0.41	0.20	2.07	0.038
Density of Neurologists	0.47	0.21	2.22	0.027
Overcoming barriers (B7a)	−0.51	0.22	−2.38	0.017

Markers for model comparison were indicative of similar performances in the “full model” with 32 predictors compared to the reduced one (*cf.* Supplementary Figure 1).

## 4. Discussion

In this study, we identified factors such as lower educational levels and structural obstacles or lack of support offerings as important factors contributing to insecurity and the feeling of not having received adequate health services during the COVID-19 pandemic among German PwP. To the best of our knowledge, this is the first time that variables relating to PwP perceived access to healthcare were investigated. With our study, we demonstrate that not all individuals were affected equally but that structural and individual aspects infer perceived access to healthcare. Viewing the pandemic through the focal lens of an ongoing demographic change in Western societies, our findings may render a deeper insight into how future care of PwP may be improved.

Our results substantiate that structural challenges for individuals with Parkinsonism reinforce perceived insecurity and a feeling of not obtaining the needed healthcare. The majority of predictors from the reduced model and eight predictors from the full model may be projected to the system-level “barrier” that was put forward by Zaman et al. Interestingly, good overall performance has been attested to the German healthcare system during the pandemic (27), which is transferable to PwP (12). However, a good testimony for a healthcare system should not be equated with an adequate range of services, especially when it comes to the very specific needs of PwP. In the recent literature, care deficits on a structural level have been reported in sinuating a rather partial insufficiency (28–31) for this heterogeneous population. One of the major challenges physicians face when treating Parkinsonism is its diverse clinical manifestation. Multimodal complex treatments could be a potential remedy (28), yet the limited availability of such services causes long journeys for people from some regions (28) as coordinated care approaches for PwP remain rare in some parts of Germany (29). Furthermore, staff providing specialized, structured, and cooperative care services are lacking especially in outpatient care and in nursing homes (30) despite being advisable (31, 32).

On the level of individual determinants, our data may also have some implications. We identified low educational attainment as a predictor for the perception of inadequate healthcare, which according to Zaman et al. (19) relates to two dimensions: health literacy and self-efficacy. The former inversely correlates with the ability to express healthcare needs (33, 34) and with educational levels of PwP (35). This is in good accordance with higher rates of hospitalizations and a higher caregiver burden (35), as well as higher disease severity (35), in PwP with lower health literacy. In addition, it has been recognized that higher educational levels relate to higher self-efficacy (36) and, at the same time, a better-perceived quality of life (36, 37). In line with these findings, our model suggests that PwP who have received more education and who present with higher quality of life scores show the greatest probability to absorb disruptions in healthcare. Contrarily, our data hence advocate for greater attention to PwP with lower levels of education, particularly those with quality of life restrictions.

Unsurprisingly, economic problems were highlighted in our results and are consistently cited as a reason for not seeking care services (19). Barring direct costs, e.g., those services spared from health insurance, many patients also claim indirect expenses like those resulting from the inability to work (38). This may gain importance with increases in the employment of women nowadays. In general, however, a somewhat surprising result is that women are at higher risk of perceived undersupply. The reasons are unclear, but literature indicates that women have fewer caregivers compared with their spouses (39) especially, as they are less likely to receive care from their male partners (19). A higher vulnerability to disruptions of healthcare because of the pandemic is therefore feasible and awaits future confirmation. In general, one might posit that to strengthen the resilience of Parkinsonism healthcare, strategies are needed that recognize and address both structural and individual barriers to access healthcare.

In addition to investments, reorganization, and policy reforms on the structural level (40, 41), suitable assessments may also help to make the individual needs of patients tangible (41, 42). One possible solution for subjects lacking access to healthcare services or who may not be able to ask for assistance due to insufficient health literacy could be telehealth services. These services are effective means to facilitate access to care. In this questionnaire, we could corroborate this (43, 44) as PwP familiar with telemedicine services before the pandemic reported a reduced likelihood of unmet care needs. Nevertheless, some caution is advised when interpreting these findings as this cohort must be deemed rather technology-savvy according to the nature of the questionnaire. Therefore, this process may not be generalizable for all patients (45). Further investigations are warranted, e.g., on how to increase confidence in telemedicine or how to overcome technological limitations such as high-speed Internet availability. Another possible caveat to consider is putative unintended negative effects on health equity so that PwP with low incomes or with other barriers to accessing technology could be left behind (46).

### 4.1. General limitations

At a relatively early stage and before the availability of vaccination provided some relief, our data reflect people’s unbiased and acute concerns regarding their healthcare. Despite revealing problems patients encountered during the COVID-19 pandemic, the interpretation of our results requires some caution. Hence, it was an anonymous online survey, so the representativeness of the German Parkinsonism population is not warranted. The response rate of 3% in this study was slightly lower than a comparable questionnaire study (12). As mentioned earlier, all patients filling out the questionnaire were recruited from a major patient organization in Germany. It should be noted that very few patients with advanced Parkinsonism participated in the survey. At the same time, it seems plausible that this group faced more challenges during the COVID-19 pandemic. Therefore, more in-depth research needs to be conducted for this group to be able to make statements about their care needs as well. Finally, a limitation is also the fact that the survey is based on individual information. Therefore, certain aspects cannot be verified by external sources of information (e.g., diagnosis and level of expertise of treating neurologists). Thus, these results await confirmation in observational studies with controlled demographics.

## 5. Conclusion

To learn from the pandemic in the long term, difficulties in access to healthcare must be uncovered and addressed. The results of this analysis showed that the COVID-19 pandemic did not affect all PwP equally, but that people who experienced individual and structural barriers to accessing healthcare before the pandemic were more affected. Therefore, it is important to examine these determinants more closely and to address them in future-oriented, resilient healthcare models. Further investigations into the effect of individual and structural influences, as Zaman et al. defined on measures of healthcare experiences, should be the object of further scrutiny.

## Data availability statement

The datasets presented in this study can be found in online repositories. The names of the repository/repositories and accession number(s) can be found at: https://github.com/dpedrosac/covidPD.

## Ethics statement

This study involving human participants was reviewed and approved by Ethics Committee - Philipps University of Marburg, Baldingerstraße, 35043 Marburg (Reference AZ 164/19). The patients/participants provided their written informed consent to participate in this study.

## Author contributions

TM and EC: survey conceptualization and writing, reviewing, and editing. DP and MM: patient recruitment. MM: data collection. DP, MP, and MM: data analysis plan, formal analysis, and writing the original draft preparation. DP: resources, visualization, and supervision. All authors contributed to the article and approved the submitted version.

## Funding

This survey and analysis were conducted as part of the research project “iCARE-PD” (Ref 01ED1904A). This is an EU Joint Programme–Neurodegenerative Disease Research (JPND) project. The project is supported through the following funding organizations under the aegis of JPND, http://www.jpnd.eu (Canada—Canadian Institutes of Health Research; Czech Republic—Ministry of Education, Youth and Sport of the Czech Republic; France—Agence National de la Recherche; Germany—Bundesministerium für Bildung und Forschung; Spain—National Institute of Health Carlos III; United Kingdom—Medical Research Council). MM is funded by the research project “iCARE-PD.” Funding provided by the Open Access Publishing Fund of Philipps-Universität Marburg with support of the Deutsche Forschungsgemeinschaft (DFG, German Research Foundation).

## Conflict of interest

The authors declare that the research was conducted in the absence of any commercial or financial relationships that could be construed as a potential conflict of interest.

## Publisher’s note

All claims expressed in this article are solely those of the authors and do not necessarily represent those of their affiliated organizations, or those of the publisher, the editors and the reviewers. Any product that may be evaluated in this article, or claim that may be made by its manufacturer, is not guaranteed or endorsed by the publisher.

## Footnotes

^1^ https://icare-pd.ca/

^2^ https://www.bbsr.bund.de/BBSR/DE/forschung/raumbeobachtung/Raumabgrenzungen/deutschland/regionen/Raumordnungsregionen/raumordnungsregionen-2017.xlsx?\_\_blob = publicationFile\&v = 3

^3^ https://gesundheitsdaten.kbv.de/cms/html/16402.php

^4^ https://www.suche-postleitzahl.org/downloads

^5^ https://github.com/kassambara/rstatix/
