# Supplementary material for: Impact of the COVID-19 pandemic on perceived access and quality of care in German people with parkinsonism
Source: Front Public Health. 2023 Apr 14;11:1091737. doi: 10.3389/fpubh.2023.1091737 (PMC10140578; doi:10.3389/fpubh.2023.1091737)
Supplement: Supplementary file 1 [file Data_Sheet_1.docx]

Supplementary Material

**Supplementary Table 1**: Matching of items in the questionnaire to the categories from the work of Zaman et al.

| **Question from Covid-Survey** | **Representative for** |  |
| --- | --- | --- |
| 1. A2, B1a, D7 | Autonomy | **Person-Level**  **Barriers** |
| 1. A1, A4, vWEI, B16a | Health Status |  |
| 1. D8 | Health Literacy |  |
| 1. B3, B5 | Health Belief |  |
| 1. B14 | Communication (personal) |  |
| 1. PDQ-8 | Self-efficacy |  |
| 1. B7a, B9a/b | Transportation |  |
| 1. B11, B12, B13, D9, D10 | Cost of care |  |
| 1. D2 | Other |  |
| 1. NA | Difficulties of Diagnosis | **System-Level**  **Barriers** |
| 1. B6a | Coordination in care |  |
| 1. B15, C2c2 | Communication (system) |  |
| 1. B7, B9b, B10, C3_3, nPop, nGer | Disparity in Health Services |  |
| 1. B6, B7a, B9, B9a, C2, C2c2 | Unavailability of Specialist Services |  |

**Supplementary Table 2**: Odds ratios for the distinct items of the questionnaire

| **Factors** | **Domain** | **OR** | **CIlow** | **CIup** | ***p*** |
| --- | --- | --- | --- | --- | --- |
| A1 - Disease duration [Years] | Health Status | 0.9 | 0.69 | 1.16 | 0.419 |
| A2 - Disease Stage [H&Y] | Autonomy | 1.13 | 0.87 | 1.47 | 0.367 |
| A4 - Presence of comorbidities | Health Status | 1.26 | 1.06 | 1.49 | 0.007 |
| B1a - Regular caregiver present | Autonomy | 0.79 | 0.43 | 1.44 | 0.438 |
| B3 - Perceived GP expertise | Health Belief | 0.71 | 0.51 | 0.98 | 0.038 |
| B5 - Perceived Neurologist expertise | Health Belief | 0.52 | 0.39 | 0.7 | p *<* .001 |
| B6 - No. of healthcare providers  consulted pre Covid | Unavailability of Spe-  cialitsts Services | 1.24 | 0.77 | 1.99 | 0.374 |
| B6a - Perceived cooperation be-  tween healthcare providers | Coordination in Care | 0.99 | 0.66 | 1.49 | 0.975 |
| B7 - Presence of geographical bar- riers in access to healthcare pre  Covid | Disparity in Health Ser- vices | 1.9 | 1.08 | 3.33 | 0.026 |
| B7a - No. of structural and trans-  portation resources against geo- graphical barriers pre Covid | Unavailability of  Specialists Services/ Transportation | 2.27 | 0.79 | 6.51 | 0.129 |
| B9 - Not received needed healthcare  pre Covid | Unavailability of Spe-  cialits Services | 2.5 | 1.88 | 3.32 | p *<* .001 |
| B9a - Availability of PD-specific community resources | Unavailability of  Specialists Services/ Transportation | 2.33 | 1.65 | 3.31 | p *<* .001 |
| B9b - No. of structural and trans- portation barriers in access to health-  care pre Covid | Disparities in Health- care Services/ Trans-  portation | 1.98 | 1.01 | 3.89 | 0.048 |
| B10 - Perceived difficulty of access-  ing healthcare pre Covid | Disparities in Health-  care Services | 5.37 | 2.84 | 10.17 | p *<* .001 |
| B11 - Rescheduled healthcare due to  financial burden pre Covid | Cost of care | 2.11 | 1.13 | 3.93 | 0.019 |
| B12 - Extended healthcare insur-  ance | Cost of care | 0.83 | 0.46 | 1.48 | 0.521 |
| B13 - Financial burden related to  PD pre Covid | Cost of care | 2.81 | 1.42 | 5.53 | 0.003 |
| B14 - Communication challenges pre  Covid | Communication (per-  sonal) | 2.63 | 1.18 | 5.82 | 0.017 |
| B15 - Experienced stigmatization in  healthcare | Communication (sys-  tem) | 2.84 | 1.6 | 5.03 | p *<* .001 |

**Supplementary Table 2**: Odds ratios for the distinct items of the questionnaire (continued)

| **Factors** | **Domain** | **OR** | **CIlow** | **CIup** | ***p*** |
| --- | --- | --- | --- | --- | --- |
| B16a - No. of negative health conse-  quences from barriers to healthcare | Health Status | 1.18 | 0.93 | 1.48 | 0.166 |
| C2 - Availability of remote health-  care during Covid | Unavailability of Spe-  cialitsts Services | 1.91 | 1.09 | 3.34 | 0.023 |
| C2c2 - Access to telehealth technologies during Covid | Unavailability of Spe- cialitsts Services/ Communication (system) | 0.62 | 0.15 | 2.53 | 0.5 |
| C3_3 - Confidence accessing health-  care remotely | Disparities in Health-  care Services | 2.44 | 1.32 | 4.53 | 0.005 |
| D2 - Gender * | Other | 0.55 | 0.31 | 0.98 | 0.044 |
| D7 - Living independently | Autonomy | 0.75 | 0.38 | 1.51 | 0.421 |
| D8 - Education [ISCED] | Health Literacy | 0.82 | 0.58 | 1.18 | 0.284 |
| D9 - Net household income  [per/year] | Cost of Care | 1.08 | 0.67 | 1.75 | 0.75 |
| D10 - Financial stability | Cost of care | 2.43 | 0.97 | 6.09 | 0.059 |
| PDQ-8 - PDQ-8 score | Self-Efficacy | 1.03 | 1.01 | 1.05 | 0.011 |
| nPop - Population according to  quantiles of German population [in sqkm] | Disparity in Health Ser- vices | 0.96 | 0.77 | 1.19 | 0.714 |
| nGER - Neurologists nearby (per  sqkm) | Disparity in Health  Services | 0.97 | 0.87 | 1.07 | 0.527 |
| vWEI - Comorbidity Index [vWEI] | Health Status | 1.08 | 0.97 | 1.21 | 0.155 |


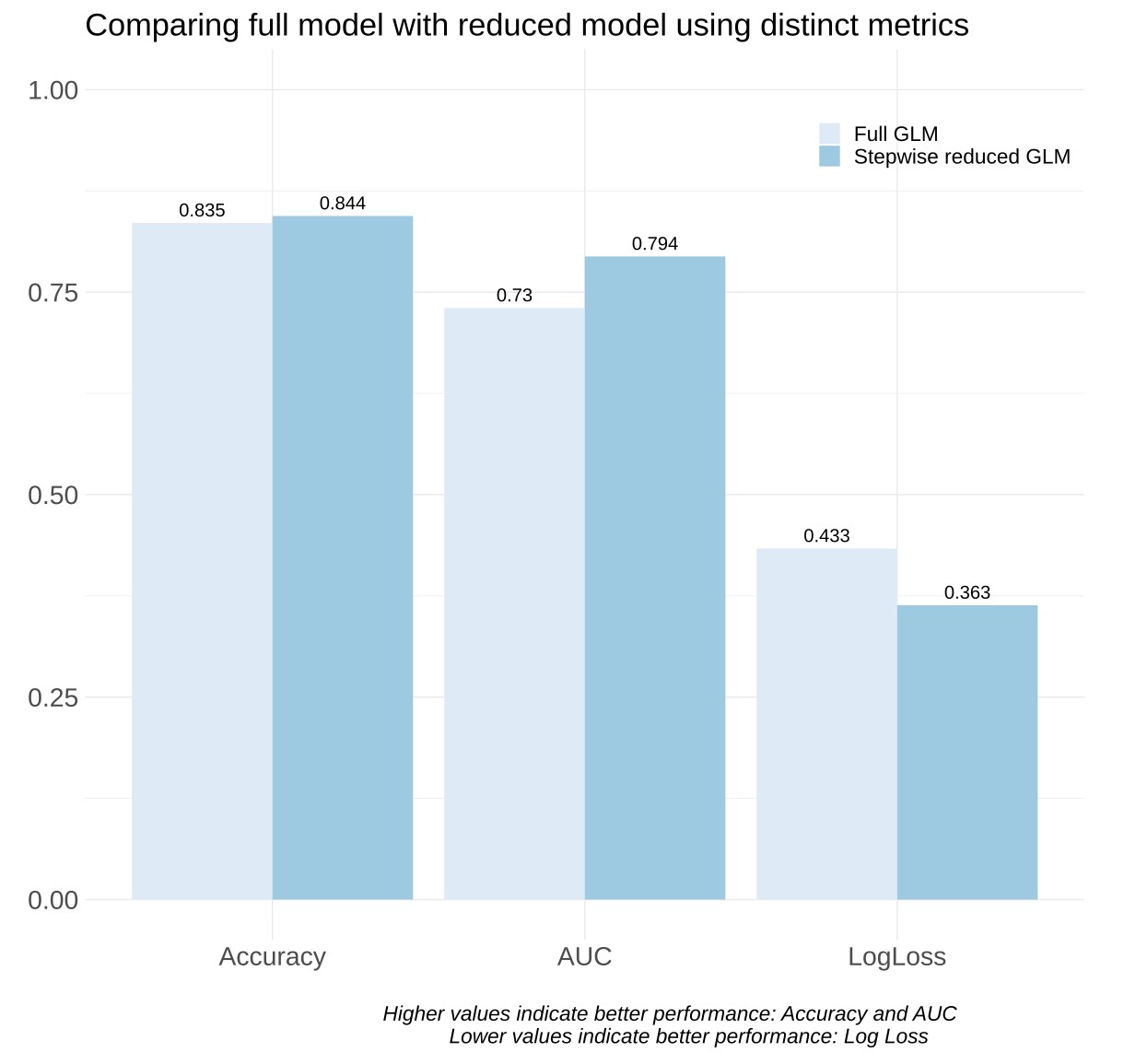


**Supplementary Figure 1**: Comparison of the models. The full model including all 32 predictors was comparted in terms of accuracy to the reduced model resulting from the stepwise GLM regression. Values between both models are comparable although only 7 predictors remained in the model compared to the full model. For further details of the multilevel regression cf. Table 2.
